# Herpes Encephalitis as a Differential Diagnosis of Atypical Intracerebral Hemorrhage: A Case Series and Systematic Review

**DOI:** 10.3390/life16061035

**Published:** 2026-06-22

**Authors:** Mark Christian Link, Judith N. Wagner

**Affiliations:** 1 Department of Neurology, Evangelisches Klinikum Gelsenkirchen, Teaching Hospital University Duisburg-Essen, 45879 Gelsenkirchen, Germany; link@evk-ge.de; 2 Department of Neurology, University Hospital Essen, University of Duisburg-Essen, 45147 Essen, Germany

**Keywords:** encephalitis, herpes simplex virus, stroke, cerebral hemorrhage

## Abstract

Herpes simplex virus encephalitis (HSVE) is the most common infectious encephalitis and is associated with high morbidity and mortality when not treated in time. Symptoms include fever, headache, fatigue, neurological deficits such as aphasia and epileptic seizures. While hemorrhagic transformation is a common complication in HSVE, intracerebral hematoma (ICH) as the initial or main presentation is rare. We present two patients with HSVE who displayed a large temporal hematoma as the main finding on cerebral imaging. We further conducted a systematic literature review to identify all published cases of ICH in HSVE. Forty-nine publications met the inclusion criteria, describing a total of 55 patients. In 38 of these, HSVE could be definitely confirmed by brain biopsy, autopsy or PCR. We analyzed these cases according to age, gender, lag from symptom onset to hospital admission, lag from hospital admission to detection of hemorrhage, location of encephalitis and hemorrhage, received treatment and outcome. With a median age of 45 years, this cohort is significantly younger than general HSVE populations described previously. In conclusion, our review shows that, albeit rare, awareness about ICH complicating HSVE is highly relevant as failure to recognize an atypical course of HVSE may result in a delay of effective antiviral treatment, which is related to an unfavorable or even fatal outcome.

## 1. Introduction

Herpes simplex virus encephalitis (HSVE) is the most frequent infectious encephalitis and accounts for about 50–70% of virus encephalitis where the virus can be identified [1]. Symptoms include fever, headache, fatigue, neurological deficits such as aphasia and epileptic seizures. HSV-PCR of CSF has become the standard for the diagnosis of HSVE, with MRI and brain biopsy in a secondary role. Before the discovery and widespread treatment with acyclovir in the 1980s, HSVE had a very high mortality of approximately 70% [2]. Even with antiviral treatment, mortality is still reported at 4–14% in various demographic studies, with a high percentage of patients (up to 50%) suffering from modest to severe sequelae [3,4,5]. Swift recognition of HSVE and timely application of acyclovir is therefore considered most important for the outcome.

While hemorrhagic transformation is a common complication in HSVE, intracerebral hematoma (ICH) as the initial or main presentation is rare. It may delay diagnosis and initiation of therapy and thereby worsen the prognosis. We present two patients with HSVE who displayed a large temporal hematoma as the main finding on cerebral imaging. We also conducted a systematic review on ICH in the wake of HSVE, describing age, gender, lag from symptom onset to hospital admission, lag from hospital admission to detection of hemorrhage, location of encephalitis and hemorrhage, received treatment and outcome. A main finding is that, with a median age of 45 years, the ICH-HSVE cohort is significantly younger than general HSVE populations described previously [6].

## 2. Materials and Methods

For the case reports, patient data were retrieved retrospectively from the electronic patient files at Evangelisches Klinikum Gelsenkirchen. Patient consent for publication was obtained. The case study was approved by the ethics committee Westfalen-Lippe, Münster, Germany (reference number 2024-240-f-S).

We conducted a systematic review of the medical literature to identify all published cases of hemorrhages in HSVE using MEDLINE/PubMed. Search terms were “herpes encephalitis” and “hemorrhage”. To get the most complete list of cases possible, the study period was not limited and there were no restrictions as to language and age of patients. Articles in languages other than German or English were included and translated using Google Translator (Version 10.22.37.928447121.0).

The inclusion criteria for final analysis were (1) cases diagnosed as HSVE and (2) radiological evidence of HSVE-associated non-petechial ICH by computed tomography (CT) or magnetic resonance imaging (MRI). Cases diagnosed by PCR of CSF, brain biopsy or autopsy material were considered confirmed HSVE, while cases diagnosed by serology or clinical characteristics were categorized as probable. Duplicate studies, preclinical studies, editorials and reviews were excluded except for secondary search. No further exclusion criteria applied. We analyzed the cases according to age, gender, lag from symptom onset to hospital admission, lag from hospital admission to detection of hemorrhage, location of encephalitis and hemorrhage, treatment and outcome. The two cases reported here were included in the overall analysis. Cases with confirmed HSVE and related ICH were analyzed separately from those with either probable HSVE and/or ICH that is possibly unrelated to the encephalitis.

## 3. Results

### 3.1. Case Reports

#### 3.1.1. Case 1

**Figure 1:**
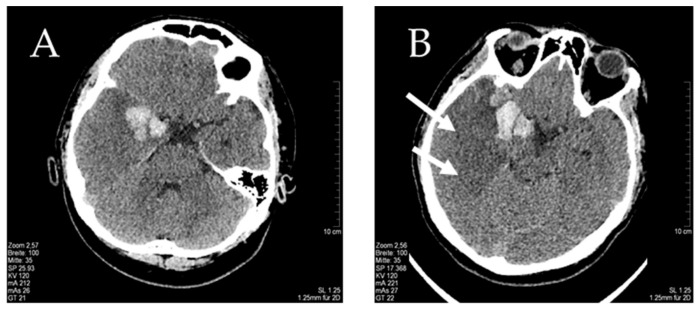
(**A**) CT of patient 1 showing several closely grouped right temporo-basal hemorrhages on day of admission. (**B**) CT control 2 days later showing markedly increased brain swelling (white arrows).

In February 2016, a 37-year-old male patient of Turkish origin was admitted with strong persistent holocephalic headache. The headache was reported to have started 14 days before with an acute onset. The neurological examination showed no deficits and the patient was somnolent but sufficiently cooperative. Body temperature was unremarkable; fever in the two weeks before admission was not reported. The laboratory results showed slightly elevated leukocytes (11.92/nL) and a mildly increased CRP (2.9 mg/dL). Further laboratory results were inconclusive; lumbar puncture was not performed. The computerized tomography (CT) scan revealed several closely grouped intracerebral hemorrhages in the right temporal lobe, the largest with a diameter of 2.4 cm (Figure 1A). Neither CT angiography (CTA) nor digital subtraction angiography (DSA) showed signs of venous sinus thrombosis, aneurysms, artero-venous malformation or vasculitis. Two days later, the patient’s level of consciousness deteriorated. The CT showed a large edema of the right hemisphere that was initially interpreted as a cerebral infarction (Figure 1B). Because of increased intracerebral pressure with midline shift the patient was transferred to the department of neurosurgery for hemicraniectomy. After surgery he remained on the intensive care unit and died two weeks later without having regained consciousness. At autopsy, a massive HSV encephalitis was determined as the cause of death. No further causes for the neurological deterioration or the ICH were specified.

#### 3.1.2. Case 2

In 2024, a 49-year-old female Turkish patient presented with a three-day history of subfebrile temperature (38.4 °C) and refractory holocephalic headaches (Visual Analog Scale, VAS 4 out of 10). The neurological examination was unremarkable. The cranial computed tomography (CT) showed a right frontal defect after surgery for an oligoastrocytoma several years before. It did not reveal any edema or hemorrhage. Laboratory results were normal apart from a slight leukocytosis (12.31/nL). The patient was admitted to our neurological department, where she developed herpes labialis. The patient refused lumbar puncture. Because her symptoms pointed to HSVE, acyclovir 3 × 750 mg was started intravenously and oralized at discharge four days later. At this point she was free of pain.

**Figure 2:**
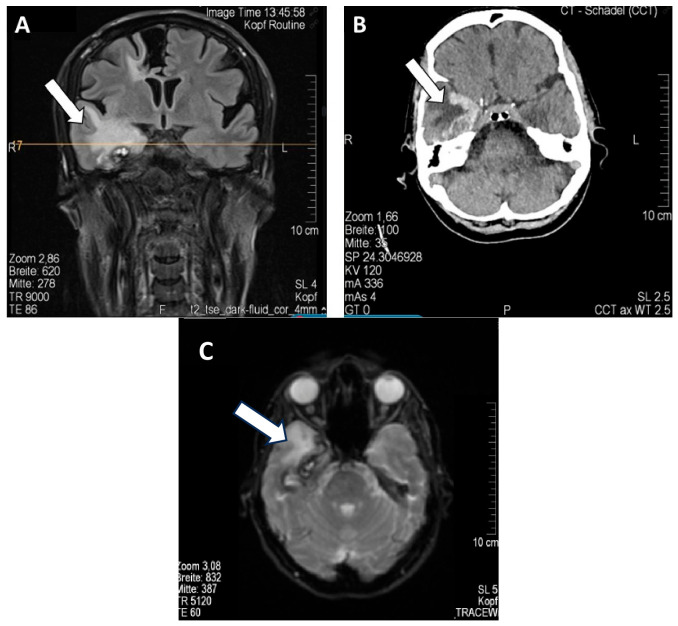
FLAIR-weighted MRI of patient 2 showing a right temporal hyperintensity as a correlate of HSVE ((**A**) arrow). Cranial CT shows a right temporal hyperdensity (hemorrhage) within a hypodense region correlating to cerebral edema ((**B**) arrow). DWI-weighted MRI demonstrates diffusion restriction within the lesion ((**C**) arrow).

Sixteen days later, the patient presented with an exacerbation of her headaches (VAS 5) and hypertensive dysregulation. The neurological examination remained unremarkable. The temperature was again subfebrile at 38.0 °C. However, the cranial CT scan showed intracerebral hemorrhage in the right temporal lobe (Figure 2B). CT angiography was normal. Lumbar puncture revealed CSF pleocytosis (78 cells/µL, 95% mononuclear cells) and CSF-specific oligoclonal bands. PCR for herpes virus type 1 and 2 had a negative result (Altostar^®^ real-time PCR reagents, manufactured by altona Diagnostics, 22,767 Hamburg, detection rate 34 copies/mL for HSV1 and 73 copies/L for HSV2), but HSV IgG in the CSF were drastically elevated with an ASI of 84.62. The MRI showed an extensive temporal edema and restricted diffusion, compatible with subacute herpes encephalitis (Figure 2A). The patient was treated with i.v. acyclovir 3 × 750 mg and discharged in regular general and neurological condition 16 days later. At discharge she was free of pain and fever. We diagnosed her with intracerebral hemorrhage due to herpes encephalitis.

### 3.2. Systematic Literature Review

**Figure 3:**
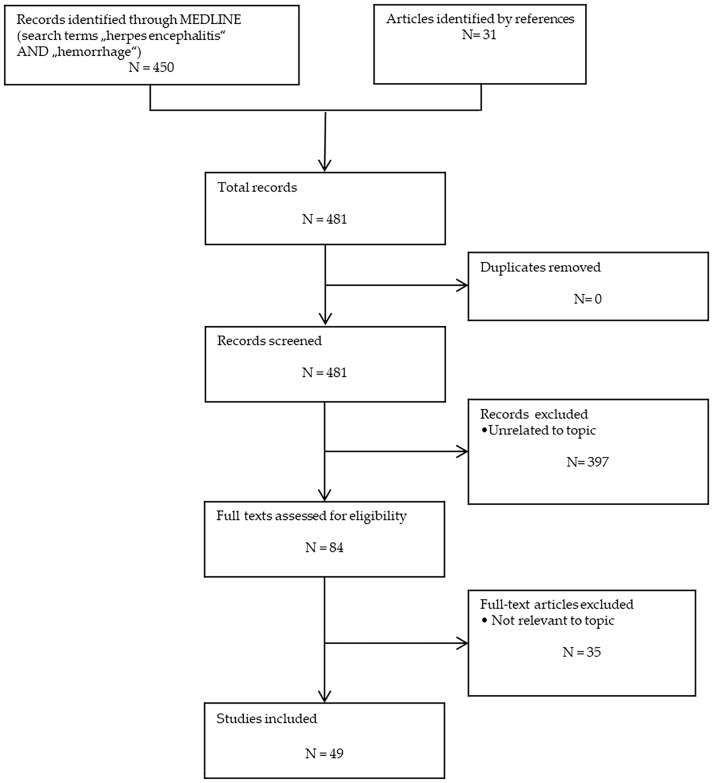
Flow chart of literature review.

A total of 450 articles were identified by Pubmed search (Figure 3). Of these, 49 reports describing a total of 55 patients met the inclusion criteria. In 38 out of 55 patients HSVE was diagnosed by brain biopsy, autopsy or CSF PCR and, therefore, was considered confirmed HSVE. The remaining 17 cases were either considered as probable but not confirmed HSVE as direct virus detection was either not attempted or unsuccessful or they were diagnosed as HSVE, but ICH could be explained by other causes:

In an older case (1980), viral encephalitis was histologically diagnosed at autopsy and HSVE was suspected on grounds of being the most frequent cause of viral encephalitis [7]. In four older cases presented between 1988 and 1995 and one case presented in 2001, the diagnosis was made on clinical and serological grounds alone [8,9,10,11]. In another two patients with negative PCR, HSVE was determined as the cause of encephalitis because of clinical criteria ([12], Link 2026). In one case the negative PCR could be explained by a previous course of acyclovir and long latency between the onset of symptoms and spinal tap as HSV-PCR frequently becomes negative within three weeks after symptom onset [13].

In one recent report a patient presented with symptoms of encephalitis, but HSV-1 was only confirmed in peripheral blood and bronchoalveolar lavage fluid [14]. One patient was diagnosed as acute hemorrhagic encephalitis (AHE), a rare and usually fatal demyelinating encephalitis, often triggered by infection [15]. Detection of HSV antibodies in CSF and a marked improvement after being started on acyclovir make HSVE more plausible in retrospect. Five patients were diagnosed with HSVE, but ICH could be explained by other causes (a newborn with disseminated intravascular coagulation, another newborn with thrombozytopenia due to rhesus incompatibility, a male patient of 63 years suffering from ICH after application of nadroparin, another patient suffering an ICH after being started on intravenous heparin because of pulmonary embolism, and a 66-year-old patient with an acute intraventricular bleeding and hepatic failure due to toxic hepatopathy) [16,17,18,19,20,21,22]. The remaining two cases represent infections with HSV-2 causing large vessel vasculitis resulting in multiple infarctions, probably with secondary hemorrhagic transformation [23,24].

For details on both cohorts see Supplementary Material.

The median age of the 38 confirmed cases was 45 years (interquartile range (IQR) 32–56). Four patients (10%) were younger than 18 years. Seventeen patients (45%) were male. The median age of the 17 cases with probable HSVE or ICH possibly unrelated to HSVE was 27 years (IQR 2–54) and 5 patients (33%) were younger than 18 years. Twelve of these patients (71%) were male.

Most hemorrhages in the confirmed HSVE cohort developed in the frontal and temporal lobe (79%), followed by the parietal and occipital lobe. Brain hemorrhage was detected on the first neuroimaging in 14 patients (37%), while 24 patients (63%) developed ICH after admission and initiation of antiviral therapy. The median time lag from symptom onset to detection of hemorrhage was 12 days (interquartile range, IQR 5–15). In 19 out of 24 patients (79%), renewed imaging was performed due to clinical deterioration. The latter comprised new focal neurological symptoms (10 patients, 42%), disorders of consciousness (5 patients, 21%), persistent/increasing headache (3 patients, 12%) and epileptic seizures (1 patient, 4%). In 5 patients (21%), it was not possible to determine from the report which symptom led to the second imaging.

In the cohort with probable HSVE or possibly unrelated ICH, only eight cases of hemorrhages were detected in the frontal and temporal lobe (47%), with ICH in the parietal lobe (three cases, 18%) and thalamus (two cases, 12%) being less common. Four patients suffered massive or multiple ICH, with two of those being newborn. The median time lag from symptom onset to detection of hemorrhage was 10 days (interquartile range (IQR) 6–12), but in six cases exact information was lacking.

All but four patients in the confirmed cohort received acyclovir (treatment not specified in one patient). All four patients who did not receive virostatic medication died. Hematoma evacuation was performed in 19 cases (50%). Outcome was favorable (modified Rankin Scale 1–2) in 44% of cases and unfavorable (modified Rankin Scale 3–5) in 32% of cases, and 24% of the patients died (Table 1). Hematoma evacuation was performed in only two cases (12%) in the second cohort. Outcome was favorable (modified Rankin Scale 1–2) in 41% of cases and unfavorable (modified Rankin Scale 3–5) in 35% of cases, 24% of the patients died.

## 4. Discussion

While hemorrhagic transformation is a common complication in HSVE, intracerebral hematoma as the initial or main presentation is rare. An analysis of the Nationwide Inpatient Sample (NIS) and Kids Inpatient Database (KID) in the United States established 4871 cases of HSVE in the United States between 2002 and 2014, with about 2.7% of the cases complicated by ICH. From the obtainable data, it was not possible to distinguish ICH and petechial hemorrhages [3]. The pathophysiology of ICH in this context is most likely multifactorial.

Atypical hemorrhage of different etiology and hemorrhagically transformed ischemic strokes constitute important differential diagnoses. In the latter, there would be corresponding CT/MRI changes limited to or most prominent in the anatomical area of the affected vascular supply. More than 60% of hemorrhages occurred after the diagnosis of HSVE had already been made. A coincidental, non-related atypical hemorrhage at the same time as the encephalitis would be highly unlikely; hence, we inferred causality. In those patients with an HSVE diagnosis post-hemorrhage, the combination of symptoms of encephalitis (fever, headache and alteration of consciousness) and neurological symptoms (seizures and focal neurological deficits) facilitated the diagnosis of encephalitis. While focal neurological symptoms could be caused by any brain lesion (including ICH), fever, rapidly developing headache and an unproportional alteration of consciousness in case of atypical ICH would make HSVE a liable explanation. Furthermore, in 79% of patients analyzed for this paper, the location of the hemorrhage conformed to the fronto-temporal HSV predilection site. The presence or absence of large edematous lesions do not seem to add to the sensitivity or specificity of the diagnosis.

Albeit rare, awareness about ICH complicating HSVE is highly relevant:

(1) Physicians treating a patient for HSVE must be vigilant and actively look for ICH in the case of a secondary deterioration despite a good initial response to acyclovir.
(2) Failure to recognize an atypical course of HVSE may result in a delay of effective antiviral treatment, particularly if cardinal symptoms such as fever and headache are absent.

Fever was described in only 71% of the reviewed patients. However, underreporting is possible. Reasons for absence of fever could be treatment with pain relievers that lower body temperature (non-steroidal anti-inflammatory drug, Paracetamol and Metamizol) or immunosuppression. None of this could be identified in our case.

While hemorrhagic transformation of brain tissue in HSVE is frequently seen, little is known about the pathophysiology of the rarer large intracerebral hematomas. Various explanations have been proposed:

(1) Small vessel vasculitis: Politei et al. reported fibrinoid necrosis in the evacuated hematoma in their case and suggested a virally induced small vessel vasculitis. This may partially explain the positive effect of corticosteroids on the outcome in HSVE [25].
(2) Massive brain tissue inflammation with collateral blood vessel affection.
(3) Increased intracranial pressure (ICP) as an indirect cause of ICH, e.g. via ischemia of arterial walls or venous congestion.
(4) A combination of some or all of these mechanisms.

At 45 years, the median age of the patients with confirmed HSVE and associated ICH is younger than in other large cohorts (57–64 years) including all cases of HSVE [3,6]. On visual inspection, the age distribution in our cohort did not suggest a clear second peak as has been described by others. A potential reason for a seemingly higher prevalence of ICH in HSVE in the young may be a more pronounced immune reaction in younger patients resulting in a higher incidence of vascular damage. Compared to these patients, those in the second cohort (probable HSVE/possibly unrelated ICH) were younger, with four of them being infants. They also presented with significantly less hemorrhages in HSVE predilection sites, which could point to a different ICH pathophysiology. We detected no sex preponderance in our cohort, which is in line with these former studies.

We found reports of HSVE complicated by ICH from all over the world (UK, France, Germany, The Netherlands, Belgium, Spain, USA, Canada, Argentinia, Peru, Tansania, Senegal, Oman, India, Japan, China and Taiwan). The differences in absolute numbers probably represent the more active scientific communities in the USA, Europe and Japan rather than an ethnic/genetic predisposition for this rare complication. Nevertheless, the small numbers do not allow for statistical analysis.

The course of the disease showed great variability in the cases included in our review. Onset was gradual in most cases, with a secondary marked deterioration of conscience and/or neurological status that led to the detection of ICH on imaging. This caused a median time lag of ICH detection from first symptom of 12 days. In most patients (63%), acyclovir had already been started when ICH was diagnosed. We found the mortality rate to be 24% in our cohort, which is much higher than in other cohorts (4—14%; [3]). This may be explained by the co-occurrence of ICH as a life-threatening complication in its own right.

In some cases, acquired disorders of coagulation may have contributed to the development of ICH. Two cases of acyclovir-induced thrombozytopenia have been reported in the literature, but none of their patients suffered an ICH [21,22]. Furthermore, only two patients included in our cohort suffered from thrombozytopenia. At least two patients included in this review were anticoagulated (nadroparin and intravenous heparin) prior to manifestation of hemorrhage [17,21]. This may have contributed to hematoma formation. The increased risk of ICH in HSVE should be taken into consideration especially in young patients when deciding on anticoagulation.

Fifty percent of patients underwent surgical intervention for ICH. This is significantly higher than reported in other reviews but may be due to a bias as many of the included cases were presented by neurosurgeons (11/38, 29%) [26].

In conclusion, HSVE may present with ICH as the predominant or even only symptom. Albeit a rare complication, timely diagnosis and initiation of treatment is vital. The treatment consists of early installation of acyclovir with surgical decompression in case of life-threatening increased brain pressure due to ICH and/or severe edema. The use of steroids in addition to acyclovir has been tried with mixed results so, presently, no final recommendation can be given. Particular awareness is required in younger patients, who accounted for the majority of cases in this review.

To our knowledge, this is the most thorough literature review on the specific challenge of HSVE presenting with ICH. This is due to the topical nature of the study and the inclusion of papers in all languages. Shortcomings include a potential publication bias and the lack of detailed information in some reports. With ICH complicating HSVE being rare, the small number of cases does not allow for further statistical analyses. Furthermore, the study design does not permit analysis of whether ICH worsens the prognosis of HSVE. This would require a cohort study, which would be an interesting design for future analysis.

## Figures and Tables

**Table 1:** Overview of clinical and radiological findings in patients with confirmed HSVE.

Criteria	Data
Median age (IQR), years	45 (32–56)
<18 years	11% (4/38)
Male sex	45% (17/38)
Days from symptom onset to detection of hemorrhage (median, IQR)	12 (5–15)
HSV-1 (PCR of CSF or biopsy)	53% (20/38)
HSV-2 (PCR of CSF or biopsy)	8% (3/38)
HSV without specification	39% (15/38)
Pleocytosis (>4 cells/μL)	86% (25/29)
Median cell count (cells/μL, IQR, *n* = 31)	130 (20–425)
Hemorrhage on first imaging	37% (14/38)
Hemorrhage after admission	63% (24/38)
Hemorrhage within HSVE predilection sites	79% (30/38)
Atypical localization of hemorrhage	21% (8/38)
Bilateral temporal lobe HSVE	26% (10/38)
No encephalitic lesion	8% (3/38)
Evidence for vasculitis	0% (0/38)
Hematoma evacuation	50% (19/38)
Good outcome (mRS 0–2)	44% (15/34)
Unfavorable outcome (mRS 3–5)	32% (11/34)
Death	24% (8/34)

## Data Availability

Data are available upon reasonable request from the corresponding author.

## Supplementary Materials

The following supporting information can be downloaded at: https://www.mdpi.com/article/10.3390/life16061035/s1. Table S1. Characteristics of patients with confirmed HSVE complicated by ICH. Table S2. Characteristics of patients with probable HSVE and/or ICH that is possibly unrelated to the encephalitis.

## Abbreviations

The following abbreviations are used in this manuscript:

CSF	cerebrospinal fluid
CT	computed tomography
FLAIR	Fluid-Attenuated Inversion Recovery
HSV	Herpes simplex virus
HSVE	herpes simplex virus encephalitis
ICH	intracerebral hemorrhage
ICP	intracranial pressure
ICR	interquartile range
KID	Kids inpatient database
MRI	magnetic resonance imaging
mRS	modified Rankin Scale
NIS	Nationwide Inpatient Sample
PCR	polymerase chain reaction
