# Supplementary material for: Herpes Encephalitis as a Differential Diagnosis of Atypical Intracerebral Hemorrhage: A Case Series and Systematic Review
Source: Life (Basel). 2026 Jun 22;16(6):1035. doi: 10.3390/life16061035 (PMC13300976; doi:10.3390/life16061035)
Supplement: Supplementary file 1 [file life-16-01035-s001.zip › life-4294482-supplementary.pdf]

Table S1. **Characteristics of patients with confirmed HSVE complicated by ICH**

**Author (year)**	**Age (years unless specified otherwise), sex (m = male, f = female)**	**HSV type, diagnostic test, CSF cell count**	**Initial clinical presentation, other findings**	**Localization of encephalitis**	**Localization of bleeding, other characteristics**	**Time from symptom onset to admission, time from admission to detection of hemorrhage**	**Treatment**	**Outcome (mRS)**
**Counsell, C. E. (1994)**[1]	32, m	HSV-1, biopsy, 750 cells/µl	Headache, nausea, fever, disorientation, mild neck stiffness	Right frontal and temporal lobe right	Right temporal lobe	Day 4, day 10	Acyclovir, craniotomy and partial temporal lobectomy	Full recovery (0)
**Malik, A. (1997)**[2]	52,m	HSV type not specified, CSF not specified, HSVE proven in biopsy	Fever, headache, neck stiffness, desorientation	Both temporal lobes	Right insula	One month, on first imaging	Not specified	Not specified
**Plantinga, E. (2000)**[3]	36, m	HSV-1, PCR, 33 cells/µl	Headache, nausea, fever, consciousness, disorientation, mild aphasia	Left temporal, frontal, and insular areas	Left temporoparietal, hematoma, edema, midline shift	Day 4, day 6	Acyclovir for 6 days, switch to foscarnet and dexamethasone on day 6	Mild aphasia and short-term memory disturbances (3)

**Author (year)**	**Age (years unless specified otherwise), sex (m = male, f = female)**	**HSV type, diagnostic test, CSF cell count**	**Initial clinical presentation, other findings**	**Localization of encephalitis**	**Localization of bleeding, other characteristics**	**Time from symptom onset to admission, time from admission to detection of hemorrhage**	**Treatment**	**Outcome (mRS)**
**Hiyama, H. (2001)**[4]	66,f	HSV type not specified , PCR neg., biopsy, 134 cells/µl	Deterioration of consciousness and headache	Right temporal lobe	Right temporal lobe	Day 1, on first imaging	Partial temporal lobectomy, no acyclovir	Death (6)
**Yan, H.-J. (2002)**[5]	48, f	HSV-type not specified, brain biopsy	Refractory headache, loss of consciousness	Left temporal lobe)	Small hematoma left temporal lobe (day 10), additional hematoma left temporal (day 12)	Day 1, day 10	Acyclovir, temporal lobectomy	Good recovery apart from mild stutter 12 months after surgery (1)
**Erdem, G.(2002)**[6]	14 months, f	HSV-type not specified, PCR, 33 cells/µl	Seizures, lethargy, fever	Both temporal, frontal, and parietal lobes	Right temporal lobe, hematoma, edema	Day 4, on first imaging	Acyclovir	Tetraparesis (4–5)
**Politei, J.M. (2003)**[7]	69,f	HSV-type not specified, PCR pos., 20 cells/µl	Fever, confusion	Left parieto occipital lobe	Left parieto-occipital lobe	Day 4, day 2	Acyclovir, haematomarevacuation	Mild aphasia and right hemiparesis (3)

**Author (year)**	**Age (years unless specified otherwise), sex (m = male, f = female)**	**HSV type, diagnostic test, CSF cell count**	**Initial clinical presentation, other findings**	**Localization of encephalitis**	**Localization of bleeding, other characteristics**	**Time from symptom onset to admission, time from admission to detection of hemorrhage**	**Treatment**	**Outcome (mRS)**
**Biswas, A. (2004)**[8]	38, m	HSV-1, PCR, 0 cells/µl	Headache, violent behaviour	Right frontal temporal lobe	Right frontal and temporal lobe, hematoma, edema	Day 7, on first imaging	Acyclovir for 14 days	Complete recovery (0)
**Kannu, P. (2004)**[9]	9, f	HSV-type not specified, PCR, 770 cells/µl	Fever, headache, vomiting, generalized status epilepticus	Right temporal lobe (CT on day 1 unremarkable)	Right temporal lobe	Day 3, day 5	Acyclovir, temporal lobectomy	Good recovery, persistent left superior quadarantonopsia (due to temporal lobe surgery) (1)
**Sakaguchi, J. (2005)**[11]	71,f	HSV type not specified, PCR, 2 cells/µl, later 31cells/µl	Fever, headache, deterioration of consciousness, neck stiffness	Bitemporal	Right thalamus, hematoma	Day 4, on first imaging	Acyclovir, Methylprednisolon	Regained Consciousness, No follow-up
**Argyriou, A.A. (2006)**[12]	22, m	HSV-1, PCR, 425 cells/µl	Encephalitis (seizure, fever, headache, altered consciousness), neck stiffness	Left temporal lobe	Left temporal lobe, hematoma	Day 2, day 11	Acyclovir	Complete recovery (0)

**Author (year)**	**Age (years unless specified otherwise), sex (m = male, f = female)**	**HSV type, diagnostic test, CSF cell count**	**Initial clinical presentation, other findings**	**Localization of encephalitis**	**Localization of bleeding, other characteristics**	**Time from symptom onset to admission, time from admission to detection of hemorrhage**	**Treatment**	**Outcome (mRS)**
**Shelley, B.P. (2007)**[13]	26, m	HSV-type not specified, PCR positive, 130 cells/μl	Fever, headache, confusion, partial seizures	Bilateral medial temporal lobe (left > right)	Left temporal lobe, hematoma	Day 2, day 18	Acyclovir	Complete recovery (0)
**Di Rienzo, A. (2008)**[14]	60, m	HSV-1, brain biopsy, 130 cells/μl	Headache, fever, neck stiffness, complex partial seizure	Right mesial temporal lobe, in control massive brain oedema	Right mesial temporal lobe	Day 1, day 14	Acyclovir, craniectomy and removal of hematoma	No neurological deficits (20 days after surgyery) (0)
**Gkrania-Klotsas, E. (2008)**[15]	46, m	HSV-1, PCR, 0 cells/μl	Headache, fever, depersonalizati on, hallucinations	No lesion	Left parietal lobe, hematoma	Day 6, on first imaging	Ayclovir	Returned to premorbid mental condition (0–1)
**Li J.Z., (2009)**[16]	56, m	HSV-1, PCR, 30 cells/μl	Confusion, mnestic deficits, seizure, HIV positive	Left medial temporal lobe	Left temporal lobe and basal ganglia, hematoma, edema, herniation	Day 14, day 6 (no hemorrhage on CT on day 1)	Acyclovir, craniotomy and evacuation of hematoma	Mild neuropsychological deficits (2)

Author (year)	Age (years unless specified otherwise), sex (m = male, f = female)	HSV type, diagnostic test, CSF cell count	Initial clinical presentation, other findings	Localization of encephalitis	Localization of bleeding, other characteristics	Time from symptom onset to admission, time from admission to detection of hemorrhage	Treatment	Outcome (mRS)
**Fukushima, Y. (2010)**[17]	35,f	HSV-1, PCR, 1112 cells/µl (1st tap), 696 cells/µl (2nd tap)	Fever, headache nausea, reduced conciousness, neuropsychological disorder	Right frontal and temporal lobe	Right temporal lobe	Not specified, day 11	Temporal lobectomy and external decompression, acyclovir	Partial recovery with persistent mnestic deficits and left hemianopsia (3)
**Tonomura, Y. (2010)**[18]	30, f	HSV-1, PCR, 321 cells/µl	Headache, fever, neuropsychological deficits, altered mental state, seizures, neck stiffness	Bilateral (left > right) medial temporal and frontal lobes	Left amygdaloid body, later subarachnoid, and ventricular blood	Day 7, day 5 (hemorrhage 1), day 26 (hemorrhage 2)	Acyclovir for 10 days, later vidarabin for 14 days, dexamethason for 5 days, IVIG 3 days	GCS 14, responds to simple commands (5)
**Takeuchi, S. (2011)**[19]	54, m	HSV type not soecified, PCR, 86 cells/µl	Fever, hemiparesis left, confusion, seizure	Right temporal lobe	Right temporal lobe, hematoma	Day 2, day 10	Acyclovir for 14 days	Hemiparesis, mnestic deficits (3)
**Battaglia, F. (2013)**[20]	38, f	HSV-type not specified, PCR, pleocytosis	Headache, fever, hallucinations, speech disturbances	Diffuse cerebral edema on CT	Left temporal lobe, hematoma, edema with brainstem compression	Day 3, day 6	Acyclovir, craniotomy and evacuation of hematoma	Died 20 days from symptom onset (6)

**Author (year)**	**Age (years unless specified otherwise), sex (m = male, f = female)**	**HSV type, diagnostic test, CSF cell count**	**Initial clinical presentation, other findings**	**Localization of encephalitis**	**Localization of bleeding, other characteristics**	**Time from symptom onset to admission, time from admission to detection of hemorrhage**	**Treatment**	**Outcome (mRS)**
**Lo, W.B. (2013)**[21]	46, m	HSV-1, PCR, 390 cells/µl	Aphasia, headache, nausea, confusion	Left temporal lobe	Left temporal lobe, hematoma, edema, uncal herniation	Day 7, day 6	Acyclovir, craniotomy, removal of anterior temporal lobe and evacuation of hematoma	Gradual improvement, no further details reported
**Rodriguez -Sainz, A. (2013)**[22]	45, f	HSV-1, PCR, 383 cells/µl	Headache, fever, mixed aphasia	Left medial temporal lobe	Left temporal lobe, hematoma, edema, brainstem compression	Day 2, day 9 (no lesion and no hemorrhage on CT on day 1)	Acyclovir for 21 days, craniotomy and evacuation of hematoma	Residual aphasia and right-sided hemiparesis (3–4)
**Rodriguez -Sainz, A. (2013)**[22]	53, f	HSV-1, PCR, 516 cells/µl	Fever, speech impairment, memory problems, headache,n ausea	Bilateral temporal lobes and insula	Left temporal lobe, hematoma, blood in subarachnoid space and midline shift	Day 6, day 8	Acyclovir for 21 days	Mild neuropsychological deficits (2)
**Yu, W. (2014)**[23]	64, f	HSV-type not specified, PCR and brain biopsy, not reported	Headache, seizure	Bilateral temporal and frontal lobe	Right temporal lobe, hematoma, raised intracranial pressure	No details, on first imaging	Craniotomy and evacuation of hematoma	Died on hospital day 25 (6)

Author (year)	Age (years unless specified otherwise), sex (m = male, f = female)	HSV type, diagnostic test, CSF cell count	Initial clinical presentation, other findings	Localization of encephalitis	Localization of bleeding, other characteristics	Time from symptom onset to admission, time from admission to detection of hemorrhage	Treatment	Outcome (mRS)
**Zabroug, S. (2015)**[24]	28, f	HSV-1, PCR, 2 cells/µl	Headache, amnesia, anxiety, paranoia, neck stiffness; 4 month postpartum	Right temporal lobe	Right infratentorial	Not specified	Acyclovir for 15 days	Good outcome (not further specified)
**Bhagchandania, D. (2015)**[25]	23, m	HSV-type not specified, PCR, 20 cells/µl	Fever, headache, seizures, „altered sensorium“	Bilateral temporal and parietal lobe	Left temporal lobe, hematoma	Day 4, day 15	Acyclovir	Behavioral abnormality (3)
**Ramesh,V. (2015)**[26]	4 months, m	HSV-1, PCR, 10 cells/µl	Low grade fever, focal seizures	Extensive lesions in right thalamus, bilateral fronto-parietal, left temporal and right insula	Hemorrhage in bilateral postcentral gyrus, in control on day 7 significant increase in hemorrhage	Day 2, on first imaging	Acyclovir	No deficits (0)

Author (year)	Age (years unless specified otherwise), sex (m = male, f = female)	HSV type, diagnostic test, CSF cell count	Initial clinical presentation, other findings	Localization of encephalitis	Localization of bleeding, other characteristics	Time from symptom onset to admission, time from admission to detection of hemorrhage	Treatment	Outcome (mRS)
**Fisahn, C. (2016)**[27]	69, f	HSV-type not specified, brain biopsy, not reported	Stroke-like (acute onset of headache and right hemiparesis)	No loco-typico lesions	Left parietal lobe, hematoma, subarachnoid hemorrhage	Day 1, on first imaging	Craniotomy for evacuation of hematoma, decopressive craniectomy	Death (6)
**Gaye, N.M. (2016)**[28]	53, f	HSV-2, PCR, 88 cells/µl	Seizures, language impairment, fever	Left mesial temporal lobe	Left temporal lobe, hematoma, ventricular blood	Day 1, day 18	Acyclovir for 21 days	Persistent severe neuropsychological deficits (5)
**Mahale, R.R. (2016)**[29]	71, m	HSV-type not specified, PCR, 5 cells/µl	Fever, headache, altered mental status	No encephalitic lesions in MRI on admission	Left parieto-occipital and right occipital region hematoma	Day 5, on first imaging	Acyclovir and dexamethason	Improvement of consciousness , Cortical blindness improved after one month (4)
**Harada, Y. (2017)**[30]	71, f	HSV-1, PCR, 170 cells/µl	Fever, headache, nausea, altered mental status	Right anterior medial temporal lobe and insular cortex	Right temporal lobe and right basal frontal lobe, hematoma, intraventricular blood	Day 8, day 14	Acyclovir	Near complete recovery (1)

**Author (year)**	**Age (years unless specified otherwise), sex (m = male, f = female)**	**HSV type, diagnostic test, CSF cell count**	**Initial clinical presentation, other findings**	**Localization of encephalitis**	**Localization of bleeding, other characteristics**	**Time from symptom onset to admission, time from admission to detection of hemorrhage**	**Treatment**	**Outcome (mRS)**
**Mueller, K. (2017)**[31]	40, f	HSV-2, PCR, 558 cells/µl	Headache, fever, nausea, vomiting	CT on first day normal	Right temporal lobe, hemorrhage, midline shift	Day 7, day 7	Acyclovir for 21 days, hemicraniectomy and temporal lobectomy	Survived, no further details reported
**Sivasankar, C. (2019)**[32]	71, f	HSV-1, immunohistochemistry on brain biopsy, not reported	Fever, decreased responsiveness, left hemiparesis, seizure	Both temporal and parietal lobes	Right temporal lobe, hematoma, edema, uncal herniation	Day 1, on first imaging	Acyclovir (delayed), craniotomy day 8	Died on day 17 after hospital admission (6)
**Byun, Y.H. (2018)**	34,f	HSV-1, PCR, 510/µl	Fever, myalgia and severe headache	Right temporal lobe	Right temporal lobe hemorrhage	Day 14, day 4	Acyclovir and dexamethasone, hemicraniectomy	Full recovery (0)
**Cueto-Fuentes, C.A. (2020)**[33]	3, m	HSV-1, PCR, 36 cells/µl,	Fever, headache, vomiting, focal seizure)	Left occipital lobe	Left occipital lobe hematoma, 2nd hematoma left hippocampus	Day 2, on first imaging	Acyclovir, dexamethason	Death (6)

Author (year)	Age (years unless specified otherwise), sex (m = male, f = female)	HSV type, diagnostic test, CSF cell count	Initial clinical presentation, other findings	Localization of encephalitis	Localization of bleeding, other characteristics	Time from symptom onset to admission, time from admission to detection of hemorrhage	Treatment	Outcome (mRS)
**Mak, G. (2020)**[34]	36, m	HSV-2, biopsy, not specified	Headache, nausea, postural changes	Right frontal and right cerebellar, in control left cerebellar, right frontal and bitemporal	Right frontal and bicerebellar, in control 3 hemorrhages left frontal and left temporal	3 months, on first imaging	Acyclovir, decompressive hemicraniectomy	Death (6)
**Canuto, D. V. (2021)**[36]	45, f	HSV-1, PCR, not specified	Fever, headache	Right temporal lobe	Right temporal lobe, hematoma	Day 3, day 10	Acyclovir	Good outcome apart from fatigue (1)
**Erdogan, H. (2024)**[37]	52, f	HSV-1, PCR, 895 cells/µl	Headache, confusion, generalised epileptic seizure	Bitemporal	Left anterior temporal lobe	Day 2, on first imaging	Acyclovir for 14 days	Persistent neuropsychological deficits (3)
**Link, M. C. (2026)**	37,m	HSV-type not specified, autopsy	Headache, altered mental state	Right temporal lobe	Right temporal lobe	Dax 14, on first imaging	No acyclovir, hemicraniectomy	Death(6)

Table S2. **Characteristics of patients with probable HSVE and/or ICH that is possibly unrelated to the encephalitis**

**Author (year)**	**Age (years unless specified otherwise), sex (m = male, f = female)**	**HSV type, diagnostic test, CSF cell count**	**Initial clinical presentation, other findings**	**Localization of encephalitis**	**Localization of bleeding, other characteristics**	**Time from symptom onset to admission, time from admission to detection of hemorrhage**	**Treatment**	**Outcome (mRS)**
**Zegers de Beyl, J. N. (1980)**[38]	20, m	HSV-type not specified, viral encephalitis on autopsy, no PCR	Fever, headache, left hemiparesis, left hemianopsia	Biparietal	Hematoma right parietal, on control 5 days later biparietal hematoma	Day 5, day1	Not specified	Death (6)
**Mallinger, J. (1987)**[39]	20, f	HSV-type not specified, HSV-antibodies, no PCR, 315 cells/µl	Headache, fever, mild neck stiffness deterioration of consciousness, diplopia, ataxia	Right temporo-basal	Large haematoma right temporo-basal, angiom detected in biopsy	Day 4, day 8	Acyclovir, haematoma surgically removed	Good outcome (0-1)
**Schlüter, B.**	5 months, m	HSV-type not	Fever, apathy, herpes labialis	Bitemporal	Bitemporal hemorrhage	Day 2, day 3	Acyclovir, dexamethason	Good outcome (0-1)

Author (year)	Age (years unless specified otherwise), sex (m = male, f = female)	HSV type, diagnostic test, CSF cell count	Initial clinical presentation, other findings	Localization of encephalitis	Localization of bleeding, other characteristics	Time from symptom onset to admission, time from admission to detection of hemorrhage	Treatment	Outcome (mRS)
**(1991)**[40]		specified, HSV-antibodies, no PCR, 64 cells/µl						
**Schlüter, B. (1991)**[40]	3 weeks, f	HSV-type not specified, HSV-antibodies, no PCR, 80 cells/µl	Somnolence, seizures	Right hemisphere	Extensive hemorrhage right hemisphere	Day2, day 7	Acyclovir	Severe sequelae (hemiparesis, epilepsy, microcephalus, cognitive impairment) (5)
**Abzug, M. J. (2000)**[41]	Newborn, m	HSV-2, PCR positive in blood, culture positive from blood	Fever, disseminated intravascular coagulation (PTT > 106 s, TZ > 212 s)	Multiple areas of brain necrosis at autopsy (not specified)	Multiple ICH	Day 1, day 5	Acyclovir, Fresh Frozen Plasma	Death (6)

**Author (year)**	**Age (years unless specified otherwise), sex (m = male, f = female)**	**HSV type, diagnostic test, CSF cell count**	**Initial clinical presentation, other findings**	**Localization of encephalitis**	**Localization of bleeding, other characteristics**	**Time from symptom onset to admission, time from admission to detection of hemorrhage**	**Treatment**	**Outcome (mRS)**
**Yan, H.-J. (2002)**[5]	37, m	HSV-1 antibodies, no PCR, 343 cells/µl	Headache, fever, bizzare behavior, memory impairment, gustatory hallucinations, neck stiffness	Left mesial temporal	Large hematoma in left temporal lobe	Day 3, day 8	Acyclovir, temporal lobectomy	No neurological deficits (0-1)
**Siri, S. (2003)**[42]	22, m	Not specified, 82 cells/µl	Confusion, nausea, diarrhea, mild aphasia	Bitemporal	Left temporal lobe	Day 14, not specified	Acyclovir, temporal lobectomy	Persistent speech impairment and memory deficits (3)
**Jabbour, P.M (2005)[10]**	27, m	HSV-1, PCR, 189 cells/µl	Fever, seizure, headache	Right mesial temporal lobe	Right temporal lobe, hematoma, uncal herniation, and ventricular blood	Day 3, day 9	Acyclovir, craniotomy and evacuation of hematoma	No focal neurological signs (0)
**Kabakus,**	3, m	HSV-type	Fever,	Right	Left parietal	Day 10, on first imaging	Acyclovir for 14	Moderate right

Author (year)	Age (years unless specified otherwise), sex (m = male, f = female)	HSV type, diagnostic test, CSF cell count	Initial clinical presentation, other findings	Localization of encephalitis	Localization of bleeding, other characteristics	Time from symptom onset to admission, time from admission to detection of hemorrhage	Treatment	Outcome (mRS)
**N. (2005)**[43]		not specified, antibodies, 450 cells/µl	headache hemiparesis<br>Pat. was diagnosed as AHE, but diagnosis was not confirmed by brain biopsy	temporal lobe	lobe, hematoma, edema		days, dexamethason for 5 days	hemiparesis (3)
**Zepper, P. (2012)**[44]	72, m	HSV-2, PCR, 588 cells/µl, 8 days later 52/µl	Aphasia and right hemiparesis	No encephalitis, multiple infarctions in territories supplied by left MCA and right PICA, MRA showing vasculitis	Left thalamic hemorrhage	Unknown, on first imaging	Acyclovir for 14 days, piperaciilin + tazobactam	Moderate cognitive impairment, mild aphasia and right hemiparesis (3)

**Author (year)**	**Age (years unless specified otherwise), sex (m = male, f = female)**	**HSV type, diagnostic test, CSF cell count**	**Initial clinical presentation, other findings**	**Localization of encephalitis**	**Localization of bleeding, other characteristics**	**Time from symptom onset to admission, time from admission to detection of hemorrhage**	**Treatment**	**Outcome (mRS)**
**Snider, S.B. (2014)**[45]	57, f	HSV 2, PCR, 1260 cells/µl	Headache, nausea, photophobia, myalgies	Multiple infarctions in centrum semiovale and cerebellum on both sides, MRA showing vasculitis	Right thalamic hemorrhage	Unknown, 9 days	Acyclovir	Minimal deficits (1)
**van den Oever, H. (2015)**[46]	63, m	Not specified	Encephalitis, diplopia,<br>ICH after application of nadroparin	Right hemisphere	ICH intraventricular	Unspecified, day 8	Acyclovir	Death (6)
**El Shimy, G. (2017)**[47]	49, m	PCR negative, 45 cells/µl	Encephalitis (fever, headache, altered mental status)	Right temporal lobe and insula	Right medial temporal lobe, hematoma	Day 2, day 12	Acyclovir, dexamethason for one day	Returned to baseline neurological status (0)

**Author (year)**	**Age (years unless specified otherwise), sex (m = male, f = female)**	**HSV type, diagnostic test, CSF cell count**	**Initial clinical presentation, other findings**	**Localization of encephalitis**	**Localization of bleeding, other characteristics**	**Time from symptom onset to admission, time from admission to detection of hemorrhage**	**Treatment**	**Outcome (mRS)**
**Clemence, P. (2024)**[48]	Newborn, m	HSV 1/2-antibodies, no PCR, no spinal tap	Jaundice, anaemia Hb 9,6 g/dl, thrombozytopenia 30.500/µl, prolonged bleeding time, INR 1,7	Multiple lesions bifronto-temporal and right parietal	Multiple hematoma bifronto-temporal and right parietal	Day 1, not specified	Acyclovir	"Remarkable improvement" (2 weeks) (?)
**Alvarez-Perez, F. J. (2021)[35]**	66, m	HSV-1, PCR, 3 cells/µl	Headache, disorientation	Hemorrhage in CT on admission	Ventricular hemorrhage	Day 1, on first imaging	Acyclovir for 14 days, drainage of CSF and blood	Death (6)
**Wu, Z. (2026)**[49]	51, f	HSV-1 in MetaCAP, no PCR of CSF	Fever, confusion, somnolence	No lesion	Multiple biparietal lesions	Not specified, 8 days	Acyclovir and foscarnet	Persistent coma (no documentation after day 30 of admission) (5)
**Link, M.C. (2026)**	49, f	HSV PCR negative,	Fever, headache	CT on first day unremarkable,	Temporal lobe	Day 3, day 20	Acyclovir	Full recovery (0)

Author (year)	Age (years unless specified otherwise), sex (m = male, f = female)	HSV type, diagnostic test, CSF cell count	Initial clinical presentation, other findings	Localization of encephalitis	Localization of bleeding, other characteristics	Time from symptom onset to admission, time from admission to detection of hemorrhage	Treatment	Outcome (mRS)
		CSF 78 cells/µl		MRI on day 23 showing temporal lobe encephalitis				

Literature

1. Counsell, C.E.; Taylor, R.; Whittle, I.R. Focal Necrotising Herpes Simplex Encephalitis: A Report of Two Cases with Good Clinical and Neuropsychological Outcomes. *J Neurol Neurosurg Psychiatry* **1994**, *57*, 1115–1117, doi:10.1136/jnnp.57.9.1115.
2. Malik, A.; Goyal, M.; Mishra, N.K.; Gaikwad, S.B.; Padma, V. Intracerebral Haematoma Formation in Herpes Simplex Encephalitis: A Case Report. *Australas Radiol* **1997**, *41*, 303–305, doi:10.1111/j.1440-1673.1997.tb00679.x.
3. Plantinga, E.G.; Vanneste, J.A. Mild Herpes Simplex Encephalitis Worsening despite Acyclovir Treatment. *J Neurol* **2001**, *248*, 237–238, doi:10.1007/s004150170235.
4. Hiyama, H.; Tanaka, Y.; Kawakami, T.; Matsuo, S.; Sawada, T.; Hori, T.; Moriyama, T. [A case of fatal herpes encephalitis presenting massive cerebral hematoma]. *No Shinkei Geka* **2001**, *29*, 271–276.
5. Yan, H.-J. Herpes Simplex Encephalitis: The Role of Surgical Decompression. *Surg Neurol* **2002**, *57*, 20–24, doi:10.1016/s0090-3019(01)00688-7.
6. Erdem, G.; Vanderford, P.A.; Bart, R.D. Intracranial Hemorrhage in Herpes Simplex Encephalitis: An Unusual Presentation. *Pediatr Neurol* **2002**, *27*, 221–223, doi:10.1016/s0887-8994(02)00428-9.
7. Politei, J.M.; Demey, I.; Pagano, M.A. [Cerebral haematoma in the course of herpes simplex encephalitis]. *Rev Neurol* **2003**, *36*, 636–639.
8. Biswas, A.; Das, S.K.; Roy, T.; Dhibar, T.; Ghorai, S.P. Acute Intracerebral Haematoma--an Unusual Presentation of Herpes Simplex Encephalitis. *J Assoc Physicians India* **2004**, *52*, 69–71.
9. Kannu, P.; Pinnock, R. Uncommon Complication of Herpes Simplex Encephalitis. *J Paediatr Child Health* **2004**, *40*, 711–713, doi:10.1111/j.1440-1754.2004.00517.x.
10. Jabbour, P.M.; Ojemann, S.G. Herpes Simplex Encephalitis. Is Anticoagulation Safe? *Neurologist* **2005**, *11*, 187–189, doi:10.1097/01.nrl.0000159761.57148.70.
11. Sakaguchi, J.; Yonemura, K.; Hashimoto, Y.; Hirano, T.; Uchino, M. [Herpes simplex encephalitis originating from bilateral thalamic lesions with hemorrhagic component]. *Rinsho Shinkeigaku* **2005**, *45*, 368–371.
12. Argyriou, A.A.; Tsota, I.; Solomou, E.; Marangos, M.; Kalogeropoulou, C.; Petsas, T.; Dimopoulos, P.A.; Chroni, E. Intracerebral Haemorrhage as a Rare Complication of HSV-1 Meningoencephalitis: Case Report and Review of the Literature. *Scand J Infect Dis* **2006**, *38*, 63–66, doi:10.1080/00365540500264019.
13. Shelley, B.P.; Raniga, S.B.; Al-Khabouri, J. An Unusual Late Complication of Intracerebral Haematoma in Herpes Encephalitis after Successful Acyclovir Treatment. *J Neurol Sci* **2007**, *252*, 177–180, doi:10.1016/j.jns.2006.10.020.
14. Di Rienzo, A.; Iacoangeli, M.; Rychlicki, F.; Veccia, S.; Scerrati, M. Decompressive Craniectomy for Medically Refractory Intracranial Hypertension Due to Meningoencephalitis: Report of Three Patients. *Acta Neurochir (Wien)* **2008**, *150*, 1057–1065; discussion 1065, doi:10.1007/s00701-008-0019-1.
15. Gkrania-Klotsas, E.; Lever, A.M. Herpes Simplex I Encephalitis Presenting as a Brain Haemorrhage with Normal Cerebrospinal Fluid Analysis: A Case Report. *J Med Case Rep* **2008**, *2*, 387, doi:10.1186/1752-1947-2-387.
16. Li, J.Z.; Sax, P.E. HSV-1 Encephalitis Complicated by Cerebral Hemorrhage in an HIV-Positive Person. *AIDS Read* **2009**, *19*, 153–155.
17. Fukushima, Y.; Tsuchimochi, H.; Hashimoto, M.; Yubi, T.; Nakajima, Y.; Fukushima, T.; Inoue, T. [A case of herpetic meningoencephalitis associated with massive intracerebral hemorrhage during acyclovir treatment: a rare complication]. *No Shinkei Geka* **2010**, *38*, 171–176.
18. Tonomura, Y.; Kataoka, H.; Yata, N.; Kawahara, M.; Okuchi, K.; Ueno, S. A Successfully Treated Case of Herpes Simplex Encephalitis Complicated by Subarachnoid Bleeding: A Case Report. *J Med Case Rep* **2010**, *4*, 310, doi:10.1186/1752-1947-4-310.

19. Takeuchi, S.; Takasato, Y. Herpes Simplex Virus Encephalitis Complicated by Intracerebral Hematoma. *Neurol India* **2011**, *59*, 594–596, doi:10.4103/0028-3886.84344.
20. Battaglia, F.; Noudel, R.; Roche, P.-H. Herpes Simplex Virus Encephalitis Requiring Emergency Surgery. *Rev Neurol (Paris)* **2013**, *169*, 182–183, doi:10.1016/j.neurol.2012.05.010.
21. Lo, W.B.; Wilcock, D.J.; Carey, M.; Albanese, E. Neurological Picture. Herpes Encephalitis Complicated by Cerebral Haemorrhage. *J Neurol Neurosurg Psychiatry* **2013**, *84*, 1404–1406, doi:10.1136/jnnp-2013-305552.
22. Rodríguez-Sainz, A.; Escalza-Cortina, I.; Guio-Carrión, L.; Matute-Nieves, A.; Gómez-Beldarrain, M.; Carbayo-Lozano, G.; Garcia-Monco, J.C. Intracerebral Hematoma Complicating Herpes Simplex Encephalitis. *Clin Neurol Neurosurg* **2013**, *115*, 2041–2045, doi:10.1016/j.clineuro.2013.06.016.
23. Yu, W; Lee, A; Welch, B Herpes Simplex Encephalitis Presents as Large Temporal Lobe Hemorrhage. *Neurol Cases* **12014**, *1*, 12–15.
24. Zabroug, S.; Idalène, M.; Azmoun, S.; Ihbibane, F.; Tassi, N. [Postpartum herpetic encephalitis complicated by cerebral hematoma]. *Rev Neurol (Paris)* **2015**, *171*, 680–682, doi:10.1016/j.neurol.2015.03.012.
25. Bhagchandani, D.; Atam, V.; Thadani, S.; Kumar, S.; Atam, I. Acute Viral Encephalitis With Intracerebral Bleed: An Atypical Presentation. *Journal of Neurology Research* **2015**.
26. Ramesh, V.; Sankar, J. Intracerebral Hematoma in an Infant with Herpes Simplex Encephalitis. *Indian Pediatr* **2015**, *52*, 991, doi:10.1007/s13312-015-0762-3.
27. Fisahn, C.; Tkachenko, L.; Moisi, M.; Rostad, S.; Umeh, R.; Zwillman, M.E.; Tubbs, R.S.; Page, J.; Newell, D.W.; Delashaw, J.B. Herpes Simplex Encephalitis of the Parietal Lobe: A Rare Presentation. *Cureus* **2016**, *8*, e785, doi:10.7759/cureus.785.
28. Gaye, N.-M.; Grimaud, J. HSV-2 Encephalitis Complicated by Cerebral Hemorrhage in an Immunocompetent Person. *Rev Neurol (Paris)* **2016**, *172*, 169–170, doi:10.1016/j.neurol.2015.12.003.
29. Mahale, R.R.; Mehta, A.; Shankar, A.K.; Miryala, A.; Acharya, P.; Srinivasa, R. Bilateral Cerebral Hemorrhage in Herpes Simplex Encephalitis: Rare Occurrence. *J Neurosci Rural Pract* **2016**, *7*, S128–S130, doi:10.4103/0976-3147.196436.
30. Harada, Y.; Hara, Y. Herpes Simplex Encephalitis Complicated by Cerebral Hemorrhage during Acyclovir Therapy. *Intern Med* **2017**, *56*, 225–229, doi:10.2169/internalmedicine.56.7386.
31. Mueller, K.; Ryan, J.E.; Tai, A.; Armonda, R.A. Delayed Temporal Lobe Hemorrhage After Initiation of Acyclovir in an Immunocompetent Patient with Herpes Simplex Virus-2 Encephalitis: A Case Report. *Cureus* **2017**, *9*, e980, doi:10.7759/cureus.980.
32. Sivasankar, C.; White, K.; Ayodele, M. An Unusual Etiology of Acute Spontaneous Intracerebral Hemorrhage. *Neurohospitalist* **2019**, *9*, 41–46, doi:10.1177/1941874418758902.
33. Cueto-Fuentes, C.A. [Early intracerebral hemorrhage as an atypical presentation of herpes simplex-1 virus encephalitis]. *Rev Peru Med Exp Salud Publica* **2020**, *37*, 155–159, doi:10.17843/rpmesp.2020.371.4384.
34. Mak, G.; Lu, J.-Q.; de Sa Boasquevisque, D.; Perera, K. Herpes Simplex Virus Type 2 Encephalitis Presenting as Multifocal Hemorrhagic Stroke. *Can J Neurol Sci* **2020**, *47*, 563–565, doi:10.1017/cjn.2020.56.
35. Alvarez-Perez, F.J.; Paiva, F.; Lino, C.A. Intraventricular Hemorrhage as Clinical Presentation of Herpes Simplex Virus Encephalitis. A Case Report and Review of the Literature. *Int J Neurosci* **2021**, *131*, 1254–1259, doi:10.1080/00207454.2020.1787409.

36. Veiga Canuto, D.; Carreres Polo, J.; Aparici Robles, F.; Quiroz Tejada, A. Acute Cerebral Haematoma in the Course of Herpes Simplex 1 Encephalitis. A Rare Complication. *Neurologia (Engl Ed)* **2021**, *36*, 80–82, doi:10.1016/j.nrl.2019.12.002.
37. Erdogan, H.; Eltahir, M.; Ramadurai, G.; Abkur, T. Herpes Simplex Encephalitis Mimicking a Primary Intracerebral Haemorrhage. *N Z Med J* **2024**, *137*, 90–92, doi:10.26635/6965.6380.
38. Zegers de Beyl, D.; Noterman, J.; Martelart, A.; Flament-Durand, J.; Baleriaux, D. Multiple Cerebral Hematoma and Viral Encephalitis. *Neuroradiology* **1980**, *20*, 47–48, doi:10.1007/BF00346861.
39. Mallinger, J.; Schmid, M.; Neu, I.S. [Herpes simplex encephalitis with cerebral hemorrhage]. *Dtsch Med Wochenschr* **1987**, *113*, 59–61.
40. Schlüter, B.; Aguigah, G.G.; Bürk, G.E.; Horstmann, W.; Andler, W. [Herpes simplex encephalitis in childhood]. *Monatsschr Kinderheilkd* **1991**, *139*, 457–464.
41. Abzug, M.J.; Johnson, S.M. Catastrophic Intracranial Hemorrhage Complicating Perinatal Viral Infections. *Pediatr Infect Dis J* **2000**, *19*, 556–559, doi:10.1097/00006454-200006000-00013.
42. Siri, S.; Kensinger, E.A.; Cappa, S.F.; Hood, K.L.; Corkin, S. Questioning the Living/Nonliving Dichotomy: Evidence from a Patient with an Unusual Semantic Dissociation. *Neuropsychology* **2003**, *17*, 630–645, doi:10.1037/0894-4105.17.4.630.
43. Kabakus, N.; Gurgoze, M.K.; Yildirim, H.; Godekmerdan, A.; Aydin, M. Acute Hemorrhagic Leukoencephalitis Manifesting as Intracerebral Hemorrhage Associated with Herpes Simplex Virus Type I. *J Trop Pediatr* **2005**, *51*, 245–249, doi:10.1093/tropej/fmh109.
44. Zepper, P.; Wunderlich, S.; Förschler, A.; Nadas, K.; Hemmer, B.; Sellner, J. Pearls & Oy-Sters: Cerebral HSV-2 Vasculitis Presenting as Hemorrhagic Stroke Followed by Multifocal Ischemia. *Neurology* **2012**, *78*, e12-15, doi:10.1212/WNL.0b013e31823fcd4d.
45. Snider, S.B.; Jacobs, C.S.; Scripko, P.S.; Klein, J.P.; Lyons, J.L. Hemorrhagic and Ischemic Stroke Secondary to Herpes Simplex Virus Type 2 Meningitis and Vasculopathy. *J Neurovirol* **2014**, *20*, 419–422, doi:10.1007/s13365-014-0253-7.
46. van den Oever, H.; Schreurs, J.W.G.M.; Huisman, M. Life-Threatening Haemorrhage in Patients without Pulmonary Embolism Who Received Anticoagulants. *BMJ Case Rep* **2015**, *2015*, bcr2015211140, doi:10.1136/bcr-2015-211140.
47. ElShimy, G.; Mariyam Joy, C.; Berlin, F.; Lashin, W. Intracranial Hemorrhage Complicating Herpes Simplex Encephalitis on Antiviral Therapy: A Case Report and Review of the Literature. *Case Rep Infect Dis* **2017**, *2017*, 6038146, doi:10.1155/2017/6038146.
48. Clemence, P.; Mariki, H.; Mkony, M.; Manji, K.P. Neonatal Herpes Simplex Virus Encephalitis with Intracranial Bleeding in a Newborn Baby with Concurrent Rhesus Incompatibility. *BMJ Case Rep* **2024**, *17*, e255822, doi:10.1136/bcr-2023-255822.
49. Wu, Z.; Liu, F.; Wang, F.; Li, J.; Lu, H. Herpes Simplex Virus Infection With Multiple Cerebral Hemorrhages: A Case Report. *Clin Case Rep* **2026**, *14*, e71857, doi:10.1002/ccr3.71857.
